# Exploring the determinants of wellbeing in university athletes: roles of athletic identity, mental energy, and social support

**DOI:** 10.3389/fpsyg.2026.1884039

**Published:** 2026-07-29

**Authors:** Xiawei Wang, Yang Zhou, Lan Li, Qinghe Wang

**Affiliations:** 1 College of Physical Education, Nanyang Normal University, Nanyang, China; 2 School of Health Sciences, Universiti Sains Malaysia Health Campus, Kubang Kerian, Kelantan, Malaysia; 3 School of Kinesiology and Physical Education, Zhengzhou University, Zhengzhou, China; 4 Department of Physical Education, Henan University of Engineering, Zhengzhou, China; 5 College of Physical Education and Health Management, Henan Finance University, Zhengzhou, China

**Keywords:** athletic identity, mental energy, social support, university athletes, wellbeing

## Abstract

**Background:**

University athletes face psychological challenges such as role conflict, sports burnout, and emotional fluctuations while managing the demands of athletic training and academic responsibilities. These challenges can negatively affect their wellbeing, which influences athletic performance, academic adaptation, and long-term psychological health. This study aims to investigate the effects of athletic identity, mental energy, and social support on the wellbeing of university athletes.

**Methods:**

A cross-sectional design was used with 1,025 Chinese university athletes recruited via convenience sampling. Participants completed self-report questionnaires measuring social support, athletic identity, mental energy, and wellbeing. Structural equation modeling (SEM) was performed using Mplus 8.3 to examine direct and indirect relationships among these variables. Model fit was evaluated using CFI, TLI, RMSEA, and SRMR indices.

**Results:**

The final SEM showed excellent fit: CFI = 0.989, TLI = 0.988, SRMR = 0.021, and RMSEA = 0.020. Athletic identity positively predicted mental energy (*β* = 0.491, *p* < 0.001) and wellbeing (*β* = 0.113, *p* = 0.016). Social support had a strong direct effect on wellbeing (*β* = 0.435, *p* < 0.001) and indirect effects through athletic identity (*β* = 0.067, *p* = 0.016), mental energy (*β* = 0.066, *p* < 0.001), as well as a sequential mediation pathway via both variables (*β* = 0.087, *p* < 0.001). Mental energy also independently predicted wellbeing (*β* = 0.301, *p* < 0.001).

**Conclusion:**

Social support, athletic identity, and mental energy play key roles in enhancing university athletes’ wellbeing. Social support contributes to wellbeing through both direct and indirect pathways, including strengthening athletic identity and activating mental energy. The findings establish an integrated theoretical framework that offers a scientific basis and practical guidance for targeted psychological interventions aimed at improving athlete wellbeing in university sports settings.

## Introduction

The physical and psychological development of university athletes has attracted growing academic interest in recent years (Brown et al., 2022; Martin et al., 2020). Compared to their non-athlete peers, university athletes often face multiple stressors arising from the dual demands of academic responsibilities and athletic commitments (Kim et al., 2020; Li et al., 2024). These pressures pose significant challenges to their mental health and subjective wellbeing, making wellbeing a central topic in contemporary sport psychology. Wellbeing not only reflects an athlete’s psychological adaptability but also plays a crucial role in shaping their athletic performance, interpersonal relationships, and future career development (Lundqvist, 2011; Wang et al., 2025b). Therefore, a deeper understanding of the psychological mechanisms underlying wellbeing among university athletes holds both theoretical and practical value.

University athletes frequently encounter identity conflicts due to their dual roles on campus. On one hand, they are expected to meet demanding academic requirements; on the other, they must dedicate substantial time and energy to athletic training and competition. This dual-role tension can compromise psychological adjustment and reduce subjective wellbeing (Parker et al., 2021; Settles et al., 2002). According to recent developments in social identity theory, individuals construct their self-concept through social comparisons and group affiliations, particularly by evaluating their in-group relative to out-groups (Haslam et al., 2021; Haslam et al., 2020). When such comparisons yield favorable outcomes, they can enhance self-worth and perceived competence; conversely, unfavorable comparisons may induce anxiety, self-doubt, and identity fragility. Prior research has demonstrated that a strong athletic identity is associated with higher self-esteem and confidence (Cartigny et al., 2021), and can indirectly promote wellbeing by strengthening a sense of team belonging and collective support (Graupensperger et al., 2020). When athletes feel recognized and valued within their team, they are more likely to develop positive self-appraisals, thereby enhancing their psychological energy and overall wellbeing. However, athletic identity is not inherently stable; it can become vulnerable in the face of injuries, performance setbacks, or role ambiguity, potentially triggering identity crises and emotional distress (Lopes Dos Santos et al., 2020; Stokowski et al., 2022). In such instances, timely and supportive interventions from coaches, teammates, and family members can play a critical role in mitigating the negative consequences of identity disruption and fostering psychological resilience and wellbeing (Haslam et al., 2022; Hayes et al., 2023).

In exploring the psychological mechanisms underlying wellbeing among university athletes, athletic mental energy has emerged as a promising construct that offers a new perspective. The concept of athletic mental energy, as introduced by Lu et al. (2018), describes the perceived availability of cognitive, emotional, and motivational resources in sport-specific contexts. As a multidimensional psychological resource, athletic mental energy plays a central role not only in sustaining performance but also in promoting emotional regulation, psychological recovery, and overall wellbeing, particularly in high-pressure, competitive environments (Chiou et al., 2020; İslam et al., 2023; Singh et al., 2024). In the context of competitive sports, mental energy has been conceptualized as a multidimensional construct comprising psychological attributes such as sustained vitality, self-confidence, goal-directed motivation, attentional control, emotional stability, and perceived resistance to fatigue (Beedie et al., 2000; Bissett et al., 2020). These dimensions were systematically identified and quantified by Lu et al. (2018) through the development of the Athletic Mental Energy Scale (AMES), which provides an operational framework for assessing athletes’ perceived psychological energy across six empirically validated subscales. While these dimensions serve as a useful framework for empirical assessment, they also reflect broader psychological capacities that enable athletes to remain engaged, self-regulated, and adaptive in the face of ongoing demands. Empirical studies have shown that higher levels of athletic mental energy are associated with reduced anxiety and burnout, greater emotional balance, and enhanced psychological wellbeing during both training and competition (İslam et al., 2023; Singh et al., 2024; Chuang et al., 2022).

Within athletic contexts, perceived social support is widely recognized as a critical resource contributing to university athletes’ wellbeing (Freeman and Rees, 2009; Jackson et al., 2009; Simons and Bird, 2022). Social support refers to the emotional, informational, and instrumental assistance that individuals perceive as available to them in times of stress or challenge, typically originating from family members, peers, coaches, and teammates (Feizollahi et al., 2022). Perceived social support serves as a critical psychological buffer, helping athletes manage stress more effectively by enhancing adaptive functioning and psychological resilience, thereby contributing to wellbeing (Haslam et al., 2022; Rees and Hardy, 2000). Emotional support, including a sense of team belonging and recognition from coaches, contributes to emotional stability. Informational support, such as feedback and the sharing of experiences, enhances confidence and facilitates problem-solving. Instrumental support, in the form of access to resources and opportunities, helps athletes achieve their performance goals (Freeman et al., 2011). These forms of support not only contribute to enhanced athletic performance but also fulfill core psychological needs for relatedness, understanding, and assistance, thereby fostering a greater sense of meaning and life satisfaction (Rees and Freeman, 2007; Rieke et al., 2008). Empirical evidence further suggests that social support may promote wellbeing both directly and indirectly, through mechanisms such as increased self-efficacy, reduced anxiety, and improved emotional regulation (Simons and Bird, 2022; Rees and Hardy, 2000; Lu et al., 2016). Thus, examining the role of social support in the wellbeing of university athletes is essential for advancing theoretical understanding and informing supportive interventions in sport settings.

Athletic identity, perceived social support, and mental energy have been widely recognized as core psychological factors influencing wellbeing in university athletes. These variables not only function as individual predictors but may also interact dynamically through direct and indirect pathways. As a central aspect of self-concept, athletic identity reflects the degree to which individuals define themselves in terms of their role as athletes. A strong athletic identity is associated with enhanced self-worth, intrinsic motivation, and psychological belonging, which in turn contribute to greater subjective wellbeing (Graupensperger et al., 2020). Moreover, individuals with a stable athletic identity are more likely to mobilize internal psychological resources, such as confidence, emotional stability, and goal commitment—collectively referred to as athletic mental energy (Lu et al., 2018; Wu et al., 2024). When athletes experience injuries, declines in performance, or role ambiguity, collegiate student-athletes may become more vulnerable to conflicts between their dual identities as both students and athletes (Hayes et al., 2023). In such situations, they often face the combined pressures of reduced athletic performance and ongoing academic demands. Consequently, individuals may struggle to determine the relative importance and priority of these competing roles and may become uncertain about which identity should serve as the primary basis for self-evaluation. Such instability in self-evaluative standards can further weaken role clarity and reduce individuals’ capacity for psychological regulation under stressful circumstances, ultimately undermining their overall psychological adaptation and wellbeing (Hayes et al., 2023; Wu et al., 2023). External resources that reinforce role recognition and provide emotional affirmation, such as social support, may play an important role in maintaining a stable athletic identity (Graupensperger et al., 2020).

Perceived social support also plays a vital role in athletes’ psychological functioning by reinforcing identity development, enhancing internal resources, and promoting positive effects. From the perspective of social identity theory, supportive interactions with coaches, teammates, and family members reinforce one’s sense of group belonging, thereby fostering the internalization of the athlete role and contributing to a more salient athletic identity (Haslam et al., 2022). In parallel, the experience of being supported emotionally and instrumentally has been shown to facilitate the development of mental energy by meeting basic psychological needs and reducing cognitive-emotional burden during training and competition. Furthermore, social support may contribute directly to athletes’ sense of belonging and wellbeing and also be associated with psychological functioning through identity-related and motivational processes. From the perspective of social identity theory, supportive interactions with coaches, teammates, and family members can strengthen athletes’ sense of belonging to the athletic group and facilitate the internalization of the athlete role within the self-concept (Haslam et al., 2022). Previous research has shown that a salient and stable athletic identity is associated with stronger goal commitment, greater confidence, and better emotional regulation, all of which represent important components of athletic mental energy (Lu et al., 2018; Wu et al., 2024). In turn, higher levels of mental energy have been linked to more adaptive coping strategies, greater resilience under pressure, and higher levels of wellbeing among athletes (Lu et al., 2018; Chuang et al., 2022).

Mental energy, conceptualized as a multidimensional construct comprising emotional, cognitive, and motivational vitality, plays a crucial role in maintaining athletes’ psychological resilience and sustained performance. Previous research has demonstrated that higher levels of mental energy contribute to effective stress management, adaptive coping, and sustained engagement, all of which are positively associated with subjective wellbeing (İslam et al., 2023; Singh et al., 2024). For collegiate student-athletes who simultaneously face academic responsibilities and competitive sport demands, these psychological resources are particularly important for sustaining effective self-regulation and adaptive functioning under chronic performance and academic stress. Previous research has indicated that athletic mental energy is negatively associated with psychological distress and burnout, while being positively associated with wellbeing and life satisfaction among athletes (Lu et al., 2018; Singh et al., 2024). In addition, athletes with higher levels of mental energy tend to demonstrate better emotional regulation, sustained attentional focus, and stronger motivational persistence in demanding sport contexts (Lu et al., 2018). These findings suggest that athletic mental energy may function as an important pathway through which social support and athletic identity are associated with wellbeing among collegiate student-athletes.

Although social support, athletic identity, and mental energy have each been linked to athlete wellbeing, previous studies have predominantly examined these relationships separately rather than as components of an integrated explanatory framework (Graupensperger et al., 2020; Lu et al., 2018; Freeman and Rees, 2009). Consequently, limited empirical attention has been devoted to the potential mechanisms through which social support may be associated with wellbeing through the development of athletic identity and the mobilization of mental energy. In addition, Chinese university athletes are simultaneously embedded within higher education and competitive sport systems and are expected to fulfill academic requirements while maintaining regular training and competition schedules (Wu et al., 2023; Gan and Anshel, 2006). These dual demands may increase role conflict and place greater reliance on psychological resources and external support for maintaining wellbeing (Parker et al., 2021; Hayes et al., 2023). Moreover, within the collectivistic cultural context of China, social relationships and group belonging may play a particularly important role in shaping athletic identity and psychological functioning (Zhang et al., 2004; Asghar et al., 2013; Wang et al., 2025a). Examining these relationships among Chinese university athletes may extend current theoretical understanding of athlete wellbeing and provide culturally relevant evidence for psychological interventions in university sport settings.

**Figure 1:**
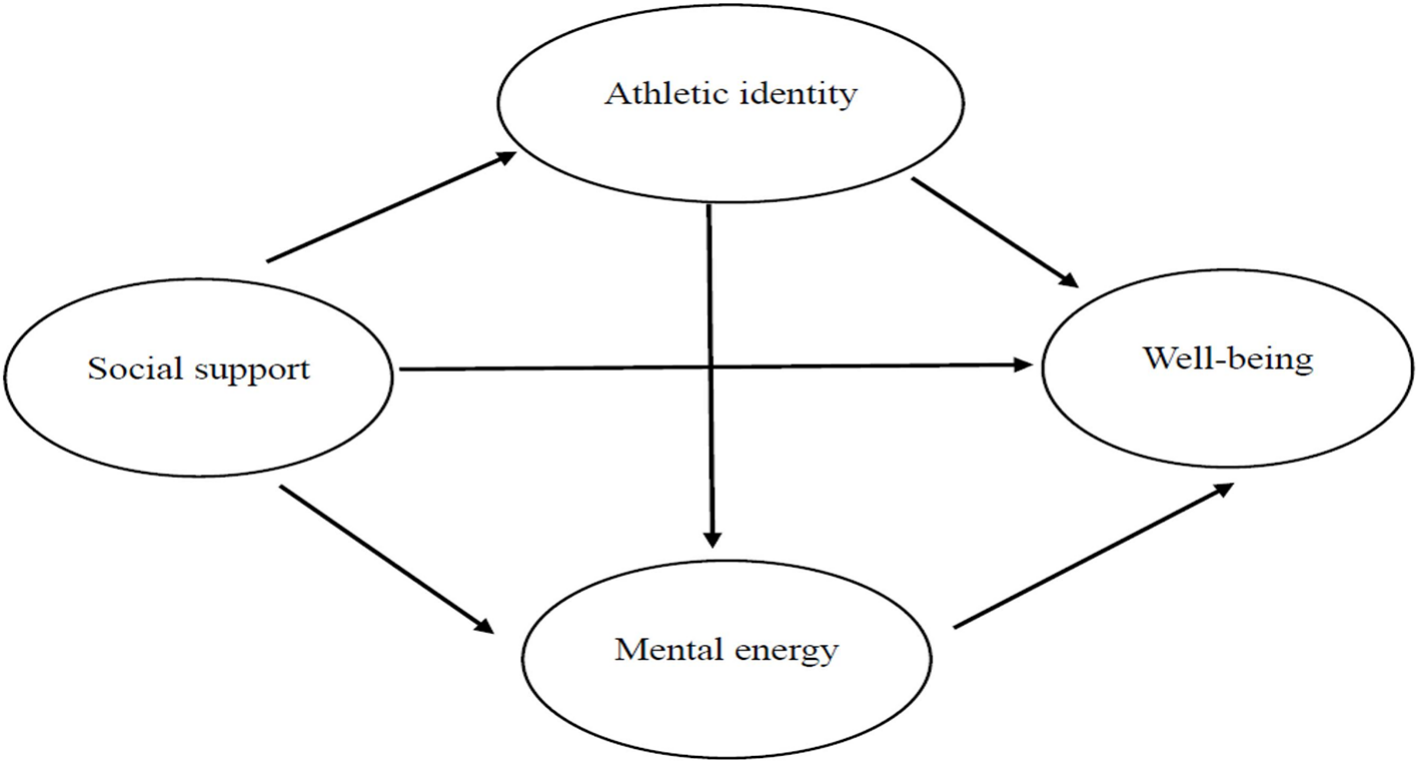
Hypothesized structural models linking social support, athletic identity, mental energy, and wellbeing.

Building upon the theoretical rationale outlined above, the present study focuses on Chinese university athletes and develops a structural equation model to examine how athletic identity, athletic mental energy, and perceived social support jointly contribute to sport-related wellbeing. The hypothesized structural model is presented in Figure 1. Based on the theoretical framework, the following hypotheses were proposed in Table 1.

**Table 1:** Hypotheses of this study.

Type	Hypothesis
Direct effects	H1: Athletic identity is related to athletic mental energy. H2: Athletic identity is related to sport-related wellbeing. H3: Perceived social support is related to athletic identity. H4: Perceived social support is related to athletic mental energy. H5: Perceived social support is related to sport-related wellbeing. H6: Athletic mental energy is related to sport-related wellbeing.
Mediation effects	H7: Athletic identity mediates the relationship between perceived social support and sport-related wellbeing. H8: Athletic mental energy mediates the relationship between athletic identity and sport-related wellbeing.
Sequential mediation effects	H9: Athletic identity and athletic mental energy sequentially mediate the relationship between perceived social support and sport-related wellbeing.

## Methods and materials

### Procedures

This cross-sectional study was conducted from August to December 2023. This study involved human participants and was approved by the Human Research Ethics Committee of Universiti Sains Malaysia (USM/JEPeM/23030250). All procedures were conducted in accordance with the ethical principles of the Declaration of Helsinki. Participants were recruited through convenience sampling from several universities in Henan Province, China. Data collection combined both online and offline surveys. For the offline survey, we visited sports teams at universities in Zhengzhou city and explained the study purpose and procedures to the athletes. The athletes participated voluntarily before or after their training sessions. Informed consent was obtained from all participants before their involvement. To ensure diversity, athletes from basketball, soccer, and athletics teams were included. To further enhance sample representativeness and reach the target sample size, an online survey was subsequently administered on the Sojump platform. Survey links and materials were shared via coaches’ WeChat and QQ groups. Coaches were responsible for providing detailed explanations of the study to their teams, ensuring that athletes had a clear understanding of the research objectives and procedures before participation. All responses were anonymous, and coaches had no access to individual responses or study data. Participation was voluntary, and athletes were informed that they could withdraw at any time without consequences. The first page of the online survey included an informed consent form. It also stated that athletes who completed the paper survey should not participate again.

A total of 1,053 questionnaires were initially collected through the combined online and offline procedures. After data screening, 28 questionnaires were excluded because of incomplete responses, duplicate participation, or response patterns indicating insufficient engagement with the survey items. The final analytic sample consisted of 1,025 valid cases. Missing data were minimal and were handled using listwise deletion. Data quality checks also included screening for straight-lining and excessively short response times in the online survey.

### Participants

This study analyzed data from 1,025 Chinese university athletes, including 653 online questionnaires and 372 offline questionnaires. Among the participants, 393 participated in team sports such as basketball, volleyball, and soccer, while the remaining 632 were involved in individual sports including badminton, table tennis, tennis, aerobics, athletics, martial arts, taekwondo, and traditional performance disciplines like dragon and lion dance. Further details on the sample characteristics are provided in Table 2.

**Table 2:** Description of the distribution of sample characteristics.

Characteristic	Frequency (n)	Percentage (%)	Mean (SD)
Sex			
Male	668	65.2	
Female	357	34.8	
Age (years)			19.9 (1.54)
BMI			21.5 (3.16)
Weekly training time (hours)			10.0 (6.14)
Years of participation in sports (years)			4.8 (2.69)
Sports level			
Ranked	464	45.5	
Non-Ranked	561	54.5	

n represents the number of categories, and % represents the percentage; M represents the mean; SD represents standard deviation; BMI was calculated as body mass index (kg/m^2^), derived from self-reported height and weight.; Ranked refers to athletes who have an officially recognized sport classification level; Non-ranked refers to athletes without an official sport classification level.

All participants met the following criteria: (1) aged 18 years or older; (2) actively enrolled in university-level athletic teams; (3) engaged in consistent training activities; and (4) qualified to compete in intercollegiate or higher-level sports events. Individuals not officially listed on team rosters or who had not participated in regular team practices over the previous 6 months were excluded.

### Measures

#### Athletic identity

Athletic identity was measured using the athletic subscale of the Chinese version of the Academic and Athletic Identity Scale (AAIS-C), originally developed by Yukhymenko-Lescroart (2014) and adapted for Chinese university athletes by Wang et al. (2025a). The subscale comprises 6 items assessing the extent to which sport participation is central to an individual’s self-concept. Participants responded on a 6-point Likert scale ranging from 1 (not central at all) to 6 (extremely central), with higher scores indicating stronger athletic identity. Confirmatory factor analysis (CFA) conducted in the present study supported the unidimensional structure, with excellent model fit: CFI = 1.000, TLI = 1.00, RMSEA = 0.000 (90% CI [0.000, 0.010]), and SRMR = 0.005. The subscale also demonstrated excellent internal consistency (Cronbach’s *α* = 0.912).

#### Mental energy

Athletic mental energy was assessed using the Chinese version of the Athletic Mental Energy Scale (AMES-C), originally developed by Lu et al. (2018) and linguistically validated by Wu et al. (2024). The AMES-C consists of 18 items measuring six psychological dimensions: vigor, confidence, motivation, tirelessness, concentration, and calmness (3 items per subscale). Participants rated each item on a 6-point Likert scale ranging from 1 (not at all) to 6 (completely), indicating the degree to which they typically experience these mental states in sport contexts. CFA results from the current sample indicated an excellent fit for the six-factor structure: CFI = 0.971, TLI = 0.963, SRMR = 0.027, and RMSEA = 0.048 [90% CI (0.043, 0.053)]. The internal consistency coefficients (Cronbach’s *α*) for the six subscales were 0.812 for vigor, 0.857 for confidence, 0.887 for motivation, 0.898 for tirelessness, 0.887 for concentration, and 0.887 for calmness. The overall scale exhibited excellent reliability (*α* = 0.930).

#### Social support

Social support was assessed using the Chinese version of the Perceived Available Support in Sport Questionnaire (PASS-Q-C), originally developed by Freeman et al. (2011) and subsequently adapted for use with Chinese athletes (Cao and Chi, 2016). The scale consists of 16 items assessing four types of support: emotional, esteem, informational, and tangible (4 items each). Responses were provided on a 5-point Likert scale ranging from 0 (*not at all*) to 4 (*extremely*), based on participants’ typical sport experiences. Confirmatory factor analysis performed in the present study demonstrated good model fit: CFI = 0.966, TLI = 0.957, SRMR = 0.036, and RMSEA = 0.052 [90% CI (0.045, 0.058)]. Cronbach’s alpha values indicated strong internal consistency for the four subscales: emotional = 0.854, esteem = 0.849, informational = 0.833, and tangible = 0.842. The overall scale reliability was excellent (*α* = 0.913).

#### Wellbeing

Sport-related wellbeing was assessed using the Chinese version of the Sport Mental Health Continuum–Short Form (Sport MHC-SF-C), adapted from the original instrument developed by Foster and Chow (2019) and validated for use with Chinese university athletes by Wang et al. (2025a). This 14-item scale evaluates mental health across three dimensions relevant to sport participation: emotional, social, and psychological wellbeing. Participants reported how often they had experienced various positive mental states during sport activities over the past month, using a 6-point frequency scale ranging from 0 (never) to 5 (every day). CFA results from the present study confirmed the three-factor structure, with good model fit: CFI = 0.962, TLI = 0.954, SRMR = 0.035, and RMSEA = 0.049 [90% CI (0.042, 0.055)]. The Sport MHC-SF-C also demonstrated high internal consistency across subscales: Cronbach’s *α* = 0.850 for emotional wellbeing, 0.875 for social wellbeing, and 0.873 for psychological wellbeing. The overall scale exhibited excellent reliability (*α* = 0.906).

### Data analysis

All statistical analyses were conducted using Mplus Version 8.3. Before model testing, data were screened for missing values and evaluated for multivariate normality. Mardia’s multivariate skewness (*p* < 0.001) and kurtosis (*p* < 0.001) tests revealed significant results, indicating a violation of multivariate normality (Kuan et al., 2019; Yao et al., 2024; Sabo et al., 2024). Therefore, the robust maximum likelihood estimator (MLR) proposed was used throughout the structural equation modeling (SEM) analysis to provide more accurate parameter estimates under non-normal data conditions (Abdullah et al., 2024; Wu et al., 2025).

SEM was employed to examine the hypothesized relationships among athletic identity, mental energy, social support, and sport-related wellbeing. A two-step modeling approach was applied: first, confirmatory factor analysis (CFA) was conducted to evaluate the measurement model; second, the structural model was tested to assess both direct and indirect pathways among latent constructs. All confirmatory factor analyses (CFAs) were specified *a priori* based on the original validated factor structures of each instrument. No *post hoc* modifications (e.g., correlated residuals, item deletions, or respecification based on modification indices) were conducted. The measurement models strictly followed the original theoretical structures of each instrument (Kline, 2023; Jing et al., 2024). Model fit was evaluated using multiple fit indices, including the comparative fit index (CFI > 0.95), Tucker–Lewis index (TLI > 0.95), root mean square error of approximation (RMSEA < 0.05), and standardized root mean square residual (SRMR < 0.08) (Wu et al., 2025; Hair, 2014; Wang et al., 2024).

To examine mediation effects, bias-corrected bootstrapping with 5,000 resamples was used. Indirect effects were considered statistically significant if the 95% confidence intervals (CI) did not include zero. The significance and strength of all effects were evaluated using standardized path coefficients (*β*), standard errors (SE), and *p*-values, with statistical significance set at *p* < 0.05. All reported path coefficients (*β*) are standardized estimates.

## Result

### Descriptive statistics and correlations

As shown in Table 3, all study variables demonstrated acceptable distributions, with skewness and kurtosis values within the recommended range. Correlation analysis revealed significant associations among all constructs in the expected directions, providing preliminary support for the hypothesized model.

**Table 3:** Descriptive statistics and correlations among study variables.

Variables	M (SD)	Skewness	Kurtosis	AMES	PASS	Sport MHC
AT	4.92 (0.80)	−0.40	−0.87	0.55**	0.50**	0.45**
AMES	4.76 (0.67)	−0.23	−0.84	1	0.42**	0.48**
PASS	3.94 (0.53)	−0.07	−1.08		1	0.51**
Sport MHC	4.28 (0.79)	−0.37	−0.77			1

M, mean; SD, standard deviation; AT, athletic identity; AMES, mental energy, PASS, social support; Sport MHC, wellbeing, *p* < 0.01 (**), two-tailed.

### Measurement and structural model

Before testing the structural model, the adequacy of the measurement model was evaluated using confirmatory factor analysis (CFA). The fit indices for each instrument are reported in the Measures section. All scales demonstrated satisfactory construct validity and internal consistency in the present sample, and all standardized factor loadings were statistically significant and exceeded recommended thresholds. These results supported the adequacy of the measurement model for subsequent SEM analyses.

### Initial structural model

**Figure 2:**
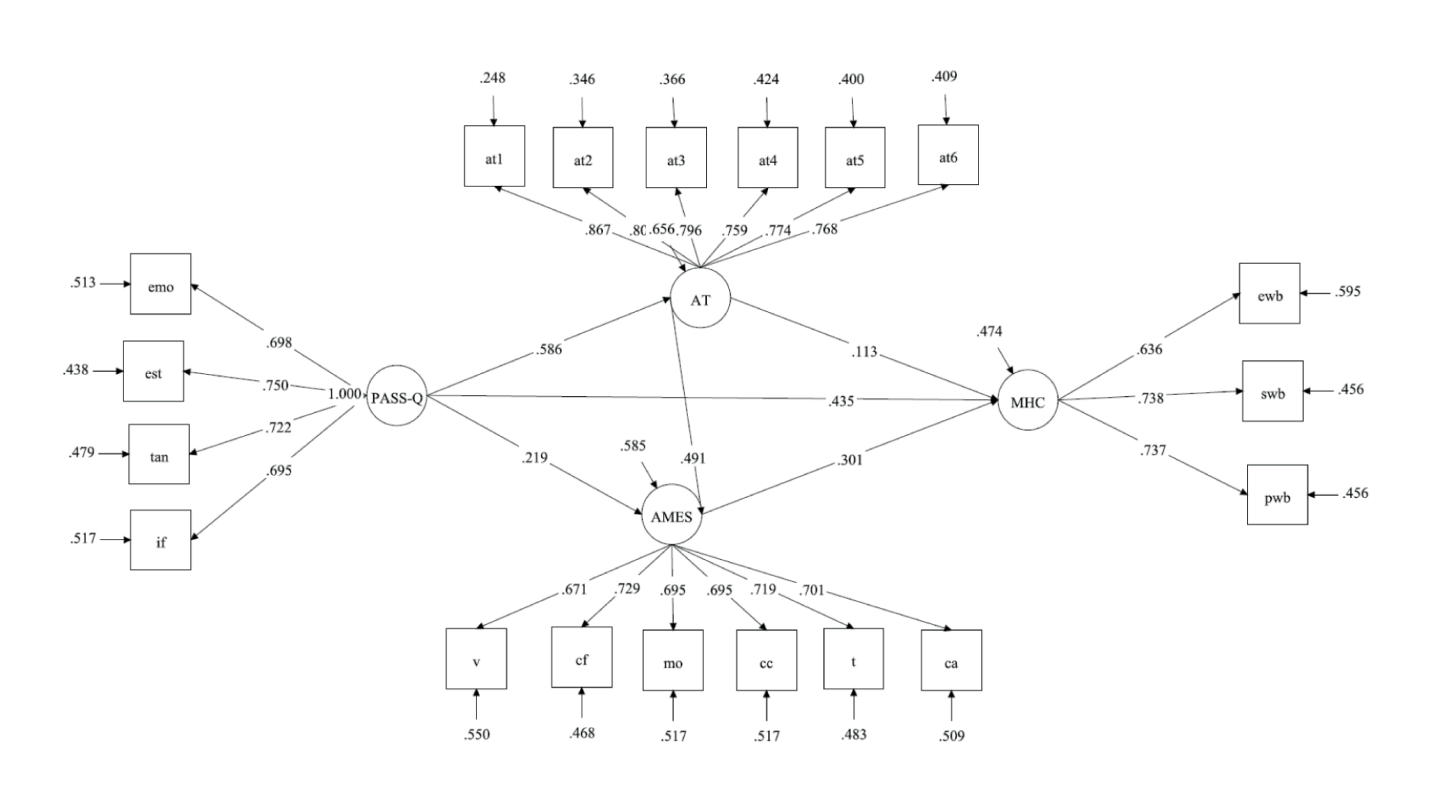
Finalized SEM model of sport support, identity, energy, and wellbeing. AT, athletic identity; AMES, mental energy; PASS-Q, social support; Sport MHC, wellbeing.

The hypothesized structural equation model demonstrated an excellent fit to the data. The fit indices were as follows: *χ*^2^ (df) = 242.648 (146), *p* < 0.001, CFI = 0.989, TLI = 0.988, SRMR = 0.021, and RMSEA = 0.020 [90% CI (0.026–0.031)]. All indices exceeded the recommended cut-off values, indicating that the proposed model provided a good representation of the observed data structure. The standardized structural model, including all latent variables and significant paths, is illustrated in Figure 2.

As shown in Table 4, all hypothesized direct paths were statistically significant. For H1, athletic identity significantly predicted athletic mental energy (*β* = 0.491, *p* < 0.001). For H2, athletic identity also had a significant positive effect on sport-related wellbeing (*β* = 0.113, *p* = 0.016). For H3, perceived available sport support strongly predicted athletic identity (*β* = 0.586, *p* < 0.001). For H4, perceived support had a significant positive effect on athletic mental energy (*β* = 0.219, *p* < 0.001). For H5, perceived support was also positively associated with sport-related wellbeing (*β* = 0.435, *p* < 0.001). Finally, for H6, athletic mental energy significantly predicted sport-related wellbeing (*β* = 0.301, *p* < 0.001).

**Table 4:** Standardized path estimates for the final structural model.

Hypothesis	Path	*β*	95%CI	SE	*z*-values	*p*-value
H1	AT → AMES	0.491	(0.428 0.553)	0.039	12.695	< 0.001
H2	AT → Sport MHC	0.113	(0.034 0.190)	0.047	2.418	0.016
H3	PASS-Q → AT	0.586	(0.537 0.628)	0.027	21.445	< 0.001
H4	PASS-Q → AMES	0.219	(0.147 0.283)	0.042	5.230	< 0.001
H5	PASS-Q → Sport MHC	0.435	(0.366 0.507)	0.043	10.174	< 0.001
H6	AMES → Sport MHC	0.301	(0.232 0.374)	0.043	7.037	< 0.001

SE, standard error; Est, estimate; *β*, standardized path coefficients; AT, athletic identity; AMES, mental energy; PASS, social support; Sport MHC, wellbeing; *z*-value, estimate divided by standard error.

In addition to the direct effects, the total, indirect, and specific indirect effects were estimated to examine potential mediating mechanisms. As presented in Table 5, the total effect of PASS-Q on sport wellbeing was substantial and statistically significant (*β* = 0.654, *p* < 0.001). The total indirect effect was also significant (*β* = 0.219, *p* < 0.001), indicating that the influence of PASS was partially mediated. Specifically, PASS had a small but significant indirect effect on sport wellbeing through AT alone (*β* = 0.067, *p* = 0.016), supporting H7. An indirect effect was also observed through AMES alone (*β* = 0.066, *p* < 0.001), supporting H8. A stronger specific indirect pathway was found through a sequential mediation of AT to AMES (*β* = 0.087, *p* < 0.001), supporting H9.

**Table 5:** Indirect and total effects from PASS-Q to MHC-SF.

Predictor variable	Through	*β*	95%CI	S.E.	*z*-values	*p-*value
Total	–	0.654	(0.609 0.699)	0.028	23.637	< 0.001
Total indirect	–	0.219	(0.176 0.263)	0.027	8.228	< 0.001
H7	via AT	0.067	(0.021 0.113)	0.028	2.409	0.016
H8	via AMES	0.066	(0.044 0.094)	0.015	4.488	< 0.001
H9	via AT→ AMES	0.087	(0.064 0.114)	0.015	5.623	< 0.001
Direct	–	0.435	(0.366 0.507)	0.043	10.174	< 0.001

SE, standard error; Est, estimate; *β*, standardized path coefficients; AT, athletic identity; AMES, mental energy; PASS, social support; Sport MHC, wellbeing; *z*-value, estimate divided by standard error.

The structural model explained 34.4% of the variance in athletic identity (*R*^2^ = 0.344), 41.5% in mental energy (*R*^2^ = 0.415), and 52.6% in sport-related wellbeing (*R*^2^ = 0.526). These results suggest that the association between perceived sport support and wellbeing operates through both identity formation and psychological energy, underscoring their critical roles as mediators in the sport context.

## Discussion

This study aimed to investigate the underlying mechanisms of wellbeing among university athletes by examining the structural relationships between athletic identity, mental energy, and perceived social support. University athletes frequently face dual pressures from academic responsibilities and competitive training, which may increase the risk of identity conflicts and psychological strain (Hayes et al., 2023; Wang et al., 2025a). Understanding how internal and external psychological resources relate to their wellbeing is thus of particular importance. Perceived social support was conceptualized as a key external factor that may influence wellbeing through internal mechanisms such as identity formation and psychological functioning. Structural equation modeling indicated significant relationships among the study variables. Notably, athletic identity and mental energy functioned as mediating variables in the association between social support and wellbeing. These findings provide new insights into the psychological adaptation process of university athletes by supporting an integrated framework in which external social resources and internal psychological mechanisms jointly contribute to wellbeing. In particular, athletic identity and mental energy appear to serve as key psychological processes linking perceived social support to wellbeing, extending the role of mental energy beyond athletic performance to psychological adaptation and wellbeing. The identified sequential pathway involving social support, athletic identity, mental energy, and wellbeing further advances understanding of how social-contextual and psychological factors may interact in shaping athletes’ wellbeing.

Firstly, the results indicated that athletic identity is significantly associated with both mental energy and sport-related wellbeing. These findings suggest that identifying as an athlete constitutes an important aspect of the self-concept, which may facilitate the development of positive psychological states and emotional experiences. Previous research has emphasized that athletic identity is a critical dimension of self-definition, contributing to enhanced self-worth, a stronger sense of belonging, and greater goal commitment (Graupensperger et al., 2020; Wu et al., 2023). When university athletes possess a clear and positive sense of athletic identity, they are more likely to maintain internal stability in the face of dual academic and athletic demands. This includes key psychological characteristics such as confidence, emotional regulation, and attentional focus (Parker et al., 2021; Abdullah et al., 2024). Furthermore, the relationship between athletic identity and wellbeing may be explained by its association with internal psychological resources such as motivation, vitality, and emotional resilience, which serve as essential foundations for maintaining wellbeing. However, previous literature also suggests that an excessively exclusive or rigid athletic identity may become maladaptive, particularly when athletes experience injury, performance setbacks, or career transitions (Hayes et al., 2023; Burns et al., 2012). Therefore, the potential benefits of athletic identity may depend on whether the athlete’s role is integrated with other important life roles, such as being a student, family member, or friend.

Secondly, perceived social support was associated with wellbeing through both direct and indirect paths. It showed a significant direct effect on wellbeing and a total indirect effect, mediated by athletic identity and mental energy. The indirect effects through athletic identity and mental energy alone were *β* = 0.067 (*p* = 0.016) and *β* = 0.066 (*p* < 0 0.001), respectively. These findings suggest that support from coaches, teammates, and family members, whether emotional, instrumental, or informational, fulfills athletes’ psychological needs for relatedness, competence, and understanding. This interpretation aligns with contemporary extensions of Self-identity Theory (Ryan and Deci, 2000), which emphasize that sustained wellbeing is associated with the satisfaction of basic psychological needs. Additionally, group identity research highlight that social affiliations in the athletic context may serve as motivational anchors that foster emotional security, role clarity, and resilience under pressure (Haslam et al., 2020). Rather than merely serving as a buffer against stress, social support is associated with the dynamic activation of internal psychological systems, thereby promoting adaptive functioning and long-term psychological wellbeing.

Mental energy emerged as a pivotal mediator linking social support and wellbeing. Although previous research has primarily focused on the role of mental energy in athletic performance and competitive outcomes, the present findings suggest that mental energy may also be relevant to athletes’ psychological adaptation and wellbeing.

Beyond its direct association with wellbeing, it served as an indirect pathway through which perceived social support was associated with wellbeing. These findings suggest that perceived social support not only enhances wellbeing directly but may also facilitate the internalization of athletic identity and the activation of psychological energy reserves, thereby promoting wellbeing more effectively. Athletes with higher levels of mental energy are more likely to experience flow states, demonstrate stable performance, and report enhanced wellbeing (Lu et al., 2018; Swann et al., 2017). For instance, Olympic athletes who exhibit high levels of confidence and concentration, core components of athletic mental energy, tend to perform better and manage stress and emotions more effectively (Arnold et al., 2018; Hägglund et al., 2022). In addition, the motivational dimension of athletic mental energy, closely related to intrinsic motivation, serves as a key psychological driver for sustained effort, goal attainment, and resilience in the face of setbacks (İslam, 2022; Lonsdale and Hodge, 2011). Regarding emotional energy, high levels of vigor and calmness have been shown to reduce athletic burnout and depressive symptoms, thereby supporting mental health (Beedie et al., 2000; Cohn and Fredrickson, 2010). As a vital psychological resource, mental energy plays a direct role in enhancing wellbeing and serves as a central mediating mechanism through which social support exerts its beneficial effects on psychological adaptation among university athletes.

Further structural modeling revealed that social support contributes to wellbeing not only through single mediation paths but also through a sequential mediating pathway involving athletic identity and mental energy. This finding suggests that social support, self-definition, and psychological resources may operate together rather than independently in relation to university athletes’ wellbeing. This finding underscores that the impact of social support on wellbeing extends beyond stress buffering or emotional relief; it also involves a dynamic psychological process that strengthens role identity and activates internal psychological resources. Social Identity Theory (Tajfel and Turner, 2004) posits that individuals who strongly identify with specific roles or groups are more likely to derive value-based motivation and sustained goal engagement. In the sports context, university athletes who experience supportive interactions with coaches, teammates, and family are more likely to construct a stable and positive athletic identity. This identity not only fosters a strong sense of role belonging but also reinforces the internal self-schema of “being an athlete. “Prior studies have consistently shown that athletic identity significantly contributes to the activation of mental energy (Cartigny et al., 2021; Gustafsson et al., 2018). Individuals with a strong athletic identity tend to demonstrate enhanced emotional regulation, sustained attentional focus, and higher achievement motivation—core elements of AMES, such as confidence, vitality, emotional stability, and fatigue resistance (Lu et al., 2018). Burns et al. (2012) found that athletes with stronger identity were more likely to gain positive experiences from social recognition and team belonging, which in turn bolstered their confidence, motivation, and emotional control, contributing to a stable level of mental energy. Social support, as an external psychological resource, serves to establish emotional affiliation and social anchoring, both of which are essential for the formation of athletic identity. A well-developed athletic identity subsequently provides a stable psychological basis for the activation and maintenance of mental energy, thereby contributing to the enhancement of university athletes’ wellbeing. Taken together, these findings support the value of considering athlete wellbeing from an integrated perspective that incorporates both social-contextual resources and internal psychological processes, particularly within the dual academic-athletic context experienced by Chinese university athletes. Coaches may help create supportive team environments that foster belongingness and confidence, while sport psychologists may focus on strengthening adaptive athletic identity and psychological resources such as mental energy. University sport programs may also benefit from providing integrated academic and psychological support services that facilitate healthy adjustment to the dual demands of sport and education.

While the proposed sequential model received empirical support, the relationships among social support, athletic identity, mental energy, and wellbeing may be more dynamic. For example, athletes with higher levels of wellbeing may be more likely to perceive support from significant others, as positive emotional experiences can broaden social engagement and shape more favorable interpretations of interpersonal interactions (Fredrickson, 2001). Likewise, those with greater mental energy may become more engaged in training and team environments, thereby reinforcing their athletic identity over time. Similarly, athletes who strongly identify with the athlete role may actively seek support or perceive social interactions more positively, given the close links between group identification, belongingness, and social connectedness (Haslam et al., 2020). These possibilities suggest that reciprocal relationships among the study variables may exist. Accordingly, future longitudinal and experimental research is needed to examine the directionality and temporal dynamics of these associations more rigorously.

Despite the valuable findings, several limitations should be noted. First, the cross-sectional design prevents conclusions about causality. All variables were assessed using self-report questionnaires collected at a single time point, which may increase the possibility of common method bias and shared variance among constructs. Although the instruments demonstrated satisfactory psychometric properties, future studies could incorporate multi-method assessments, such as coach ratings, behavioral indicators, or physiological measures, to reduce potential measurement bias. Future research should also use longitudinal or experimental designs to further examine the proposed relationships. In addition, the use of convenience sampling with Chinese university athletes may limit generalizability. Future research should include more diverse samples across regions, cultures, and sport levels. Finally, this study did not explore potential moderating factors such as gender, sport type, or training experience. Future work could use multi-group structural equation modeling to examine these differences in depth.

## Conclusion

This study investigated the associations among social support, athletic identity, mental energy, and wellbeing in university athletes. The findings indicated both direct and indirect relationships among these variables, with the strongest pathway involving a sequential association through athletic identity and mental energy. These findings suggest that external support may be linked to athlete wellbeing through pathways involving identity and internal psychological resources. This study provides further insight into the psychological processes associated with wellbeing in university athletes and highlights the potential value of integrated, resource-based approaches in psychological interventions within sport settings.

## Data Availability

The raw data supporting the conclusions of this article will be made available by the authors, without undue reservation.
